# An Account of Epidemic Metritis

**Published:** 1845-11

**Authors:** Francis H. Gordon

**Affiliations:** Wilson county, Tennessee


### Art. II.—*An Account of an Epidemic Metritis.* By Francis H. Gordon, M.D., of Wilson county, Tenn.*

The disease of which I propose in the following pages to give an account, prevailed over a limited region of country in Tennessee during the past summer and autumn. It was confined, so far as my observation extended, to the counties of

---

* The following article is a part of the Inaugural Dissertation of the writer, which was submitted to the Faculty and Managers of the Medical Institute of Louisville, at the late commencement, for the degree of M.D.

Smith and Wilson, and to a single district in these. The topography of the country cannot be said to exhibit any peculiar feature. It lies on the south side of the Cumberland river, and is drained by three small streams. The soil is calcarious, with a fertile vegetable mould, large forest trees and other luxuriant vegetation. Numerous springs of the purest crystal water abound every where, and the face of the country is sufficiently undulating to prevent large collections of stagnant water. Excepting the numerous mill-ponds on the creeks, the traveler would behold no cause of disease. Fever of a typhoid grade, and phlegmonous erysipelas prevailed epidemically from the 1st of January, 1844, till about the middle of May. In June some cases of remittent fever occurred; and from the 20th of July till frost, which was about the 25th October, remittents and intermittents were prevalent. Metritis made its appearance ten or twelve days earlier than remittent fever, and was rife till the end of October. Nor did it spare any condition or age of females after puberty. The young virgin, the pregnant and unimpregnated of meridian life, as well as the matron after the decline of the menses were all obnoxious to it.

The following cases which occurred in the practice of the writer, will show the comparative frequency of the disease, in different conditions and at different ages. There were from 14 to 20 years of age, 13 cases; from 20 to 30 years, 17 cases; from 30 to 45, 17 cases; and 6 cases over 45, making in all 53; 17 of these were unmarried, and 8 subjects were pregnant. The attack came on in three cases soon after delivery; two cases resulted in abortion, and one terminated in crural phlebitis. It is remarkable that one of the subjects of the disease was between 70 and 80 years of age. The subject of crural phlebitis was about 40, and was about 5 months gone in uterogestation when attacked; she did not abort, but had not entirely recovered when seen last on the 5th November. Three of the above cases proved fatal; and in consultation the author saw another patient who died of this disease. It is to be regretted, that there was no opportunity for post-mortem inspection of the ravages of this disease.

*Symptoms.* The attack was usually preceded by premonitory symptoms, such as a sense of weight in the pelvis, a benumbed sensation in the sacrum, thighs and legs, followed by a disagreeable tingling in the feet; to which were added, pain in head and loins, and in some instances *uterine hemorrhage.* These symptoms lasted from two to ten days, progressing from mild to severe, when others of a more formidable character came on, and prostrated the patient at once. Now there were frequent irregular rigors or distinct chills, great thirst, pyrexia, pulse frequent and hard, seldom full; tongue slightly furred and red at the tip; severe pain in the head and loins; tenderness to pressure the whole length of the vertebral column, and in one or both iliac fossæ. Upon pressure above the pubis, the fundus uteri could be felt, and lancinating pains shot up to the umbilicus, and into one or both iliac regions; restlessness, vertigo, tinnitus aurium, or partial deafness and delirium; nausea and vomiting, with pain in the stomach; urine scanty and high colored, or totally retained; constipation or tenesmus. An examination *per vaginam* excited pain in that organ and the uterus, while the sensation of great heat was imparted to the finger. The tenderness of the hypogastric and iliac regions, gradually extended over the whole peritoneum. The patient was unable to move or bear the slightest pressure without considerable pain. Tympanitis, subsultus, and jaundiced skin and eyes appeared in two of the fatal cases.

I have intentionally postponed the representation of one common symptom, viz: uterine hemorrhage. This existed in all the nongravid cases, but was not uniform as to the time of its appearance. In some instances it was premonitory, and then the disease became violent about the time, or a few days before its cessation. In most cases, the hemorrhage was simultaneous with the prostrating attack, but in a few it did not occur until the decline of the disease. In several instances where the attack supervened upon the supposed 'subsidence of the menses' the flooding came on again before recovery. Its duration was from three to ten days, and was often so profuse as to be alarming. Of the eight cases of pregnant women, two only were attended with flooding, and they resulted in abortion. Those who were attacked while nursing, suffered a suspension of the secretion of milk.

I have been thus particular in describing this feature, because it is precisely the reverse of what is common in puerperal fever, in which the lochia are usually suspended shortly after the attack.

*Treatment.*—From the long list of symptoms here given, it will be seen that there were several indications to fulfil.

1. Depletion. Where there was general pyrexia, and a sufficient vigor of the pulse in the onset, (which was not often the case) the abstraction of blood from the arm was of advantage; but when general bloodletting was thought to be unsafe, local abstraction was indicated and practised. Cups over the sacrum, loins and hypogastrium were often of benefit. But the most signal advantage was derived from bleeding in the temporal arteries: 8 to 14 ounces of blood taken in this way, relieved the congestion of the brain, and reduced the general febrile action. After a liberal bleeding of this sort, a stream of warm water was poured upon the head for one or two hours, which not only aided the lancet in relieving local congestion, but tended much to allay general irritation.

But here a short digression may be justifiable. Though local bloodletting is generally approved and practised, yet I may safely insist, that it is seldom carried to the extent which the symptoms indicate. I regard it as a good general rule to continue the abstraction from any point of congestion or inflammation till decided relief of the part is obtained, mere quantity being no criterion to govern the practice. If there be general pyrexia and a full pulse, the common rule is, to bleed from the arm, and follow it with local abstraction; but where there is considerable congestion or local inflammation, if at all practicable, I should much prefer to drain all the blood necessary to reduce the general febrile action directly from the point of suffering. Because on this plan, less blood need be abstracted to accomplish the object, both locally and constitutionally, and we are more certain of removing local hyperemia. These views governed the treatment of the disease we are considering, and there is no ground of regret on that account.

2. The second object was to clear the primaria of all vitiated accumulations, and to promote general secretion. Active purgation was contra-indicated, because it tended to produce irritation in the large intestines, and thus sympathetically to aggravate the uterine inflammation. Hence, Dover's powder or ipecac., combined with calomel and blue mass were found to answer best. These remedies were continued through the active stage of the disease; they promoted the secretion of the liver, and tended to equalize the circulation, while they kept up gentle catharsis. Where there was obstinacy of the bowels, enemata or castor oil with a few drops of laudanum were called in aid. The spirits of nitre and nitrate of potassæ were preferred as diuretics and diaphoretics.

3. The third indication was to allay nervous irritation. Dover's powder, and the salts of morphia and cicuta accomplished the object as well as could be expected from this class of remedies. The sulphate or acetate of morphia was chiefly used, and continued through the course of suffering. But the spinal cord being in a state of inflammation, or morbid irritation something more than mere palliating treatment was indispensable. The remedy relied upon, and which cannot be too highly estimated, was the unguentum antimonii, made of antimonii et potassæ tartratis ʒiii. adipis ℥i. This unguent was rubbed upon the skin over the spine and sacrum every four hours till a dense crop of pustules came out. As soon as the vesicles disappeared, the ointment was re-applied, and this treatment was continued till all tenderness to pressure, as well as all nervous symptoms disappeared. The pain in the stomach (a common attendant) was seldom more than temporarily removed by any other means; but as soon as free pustulation could be produced, the pain and other gastric distresses subsided, and seldom returned provided the vesication was continued.

4. Another object was to subdue local inflammation. So far as the foregoing treatment failed to subdue the inflammation of the uterus and its appendages, as well as the peritoneum, (and it always failed in part) local remedies became necessary. Enemata of warm water, laudanum, and starch, or of the syrup of poppies were injected into the vagina and rectum; blisters to the inside of the thighs, and poultices of wheat bran to the hypogastrium or whole abdomen. These were all of service, but warm water was the most important remedy in this part of the treatment, and the mode of applying it deserves a remark. A continued stream of warm water was poured from a pitcher upon the skin over the point of inflammation, and where the end could be accomplished, the plan was *to keep up the process till all inflammation was subdued.* The uterus and other organs deeply seated in the pelvis, could be partially, but not entirely relieved in this way. The bony structure prevented the impression from being made on the surface, directly over these organs, hence the imperfect relief here; but as to the peritoneum in the abdominal cavity, the remedy was potent. In many instances, the stream was continued for *four hours* without cessation, longer than was requisite to exchange an empty pitcher for a full one. This process subdued general febrile action, as well as local inflammation; and to avoid producing a chill, consequent to the abatement in the force of circulation thus caused, sinapisms were applied to the feet.

Having seen such manifest and speedy relief from the protracted use of the stream, in the disease under consideration as well as many other affections, the enquiry is natural: why should any limit be set to its use, so long as there remains a single vestige of local inflammation? Suppose the abdomen to be tumid, and exquisitely painful to the touch—a stream of warm water is poured upon the skin for half an hour, at the end of which, pressure is made all over the abdomen, and it is found that there is less tenderness, and that the patient can lie with the legs extended without causing pain; the benefit of the remedy being thus evinced, why discontinue it? Another half hour's trial is made, and found to be attended with still further abatement, why stop now? If one hour subdues the inflammation in part, two will do more; three still more; and if the patient's constitution can be made to bear it, I suppose there can be no good reason for discontinuing the process, till the last vestige of inflammation be removed. And the result of numerous practical trials, confirms this opinion.

5. The last indication was to support the prostrate energies of the constitution, in fulfilling which, the proper management of the concomitant uterine hemorrhage was an important object. It was regarded as nature's mode of relieving the uterine congestion, and was not therefore interferred with, unless it became profuse and prostrating. Reliance was placed upon the foregoing treatment, to restore an equilibrium of the circulation, and to improve the general health; and thereby to arrest the hemorrhage in due time. But it was sometimes found necessary to resort to prompt means. The internal use of the acetate of lead and the tampon were adequate remedies.

To sustain and revive the prostrate energies of the system the patient was allowed light but nutritious diet and wine; and quassia and quinine were administered *pro re nata.*

The treatment of stranguary, which was a very common attendant of this disease, has been postponed till now, for the purpose of noticing a new remedy, which was the almost sole reliance. I allude to an infusion of the apis mellifica, or common honey bee. Sweep 40 to 60 bees into a pan of water, so as to make them manageable; put the whole into a teacup, pour one gill of boiling water on them, and cover cup securely. When it has remained twenty minutes, pour off the infusion and let the patient take the whole at a draught. This remedy relieved the strangury in from two to fifteen minutes with great certainty.

It was introduced into the practice of medicine some years ago by Mrs. Perry—an old woman in the habitual practice of midwifery in the county of Smith; and experience proves that its efficacy is not less considerable because of its unscientific origin. Some six or seven practitioners in that section of country, (some of whom do honor to the profession) have given the remedy numerous fair trials, and so far as I have learned, all estimate it highly. The writer has tried it repeatedly in the retention of urine from inflammation of the bladder, and from the effects of cantharides, and found it to be more prompt and certain than any other remedy. There can be no question but that the "bee tea" will prove a valuable accession to our materia medica. How far it may be found to be useful in ischuria and dysuria from every variety of cause, remains to be tested; and its known value affords abundant encouragement for further investigation.

As to the class to which this agent naturally belongs, I have but little hesitation in placing it among the *narcotics.* That it acts as an antispasmodic there can be no doubt, but whether it is a specific for the sphincter muscle of the urinary bladder, as the ergot is supposed to be a specific for certain fibres. of the uterus, has not been clearly determined.

It is probable that the virus ejected by the bee in poisoning the wound it inflicts upon its enemy, is the material which gives virtue to the bee tea. This virus is secreted and collected in a sack in the abdomen of the bee, near the base of its lance or sting. When war is made upon its enemy, the virus is injected into the puncture made by the lance, and the wound is poisoned. It is also emitted during the anger of the insect, as is known by its peculiar pungent odor; and that the virus which gives out this odor is the same which imparts the antispasmodic virtue to the infusion, is evinced by two facts:

1st. The tea, when recently made, has a taste and smell identical with the odor of the incensed bee, and now the infusion is efficacious.

2d. But if the infusion be allowed to stand and cool, and especially if allowed to remain uncovered, its characteristic odor and taste disappear, and the tea is correspondingly inefficient. These facts justify another conclusion—that the virus is quite volatile, and requires care to prevent its escape.

Whether this valuable virus may not be collected and concentrated, or combined with some chemical element, so as to render it portable and convenient, is a matter of interest and well worth the attention of the chemist.

*Reflections.* In reviewing the phenomena of the foregoing disease, several enquiries are suggested, but the limits of this production will only allow a glance at some of them. Is the disease of atmospheric origin? Of the affirmative there is scarcely a doubt. The number of cases which occurred in the practice of the writer, added to those which were attended by neighboring practitioners, would probably amount to one hundred or more; all of which were included within a scope of country not more than twelve miles square. That such a number of cases, of an unusual character should occur in one season, and in so small a compass, independently of some epidemic or contagious cause, is incredible. And as to the idea of contagion, I need only remark, that there was no evidence of it. But is its remote cause the same as that of autumnal fever? An able, experienced, and scientific physician of Wilson county, inclines to this opinion. When in consultation upon one of the cases which I have termed metritis, he remarked, that autumnal fever was sometimes attended with uterine congestion and hemorrhage, and that this might be a case of that kind. To which it may be answered, that yellow fever and Asiatic cholera have also been attended with uterine hemorrhage; and so may any constitutional disease of a hemorrhagic diathesis be attended with uterine congestion and hemorrhage. But no one, I believe, will urge that such is a common result, or that it is a leading diagnostic of any of those diseases, as was the fact in the malady before us. On the contrary, it is well known that hemorrhage from the uterus in those diseases takes place not in the onset, but at the decline, and that it is merely a critical discharge from that organ.

In similar cases we have crises of sweat from the skin, of urine from the kidneys, and of blood from the nose and bowels. But in this form of metritis, we have seen that the uterine discharge was not critical in the true sense of the term. It was no evidence of change in the disease, nor was it a means by which the result might be prognosticated. So far from this was the fact, that the discharge sometimes preceded, and most commonly was simultaneous with the attack. Moreover, if this feature be a crisis, why did it not show itself in neighboring districts, where autumnal fever of the same type prevailed as was also common here? But I have been assured by several practitioners in the midst of fall fever at the time, that they did not have a single case of uterine inflammation and flooding. Again, if this was fall fever of hemorrhagic tendency, why had we no critical discharges of blood in this district except among adult females? Others had fever, and if it had been the same disease, some of them at least would have had critical discharges of blood from some organ. Indeed, I saw but one case during the whole season, that was a truly critical hemorrhage; and that was a case of epistaxis, which occurred in a young lady about the 12th day from her attack of metritis, and about two days after the cessation of profuse uterine hemorrhage.

Insomuch as erysipelas had so recently prevailed in this section, it may be well to make a remark in reference to it, because puerperal fever has been thought to be uterine erysipelas, and the same view may be taken of the cases we have reported. A deliberate consideration of the imperfect history of metritis as here given will satisfy any one on this point. But, if the characteristics of the two diseases were sufficiently analogous to render their diagnosis difficult, the history of the recent erysipelas would settle the question. It had been confined to the surface of the body and throat, and attacked both sexes, of minor as well as adult age. Hence the demand upon our credulity would be too strong, were we required to believe that the disease at once disappeared from its usual points of attack, forsaking all others of the populace and concentrating its ravages on the uterus of adult females alone.

From all of the foregoing considerations, we are brought to the conclusion that metritis, as it has now been represented, is an idiopathic as well as an endemic disease. Upon what peculiar atmospheric condition it depends is uncertain, and therefore it may well employ those who are inclined to speculate. Whether it has originated from a superabundance of carbonic acid, from animalcula infusoria, from electricity disturbed in its wonted equilibrium, from a deficient or redundant moisture, or from some unknown chemical combination or solution resulting from the spontaneous decomposition of animal or vegetable matter, must be left to the investigation of those whose curiosity may prompt, and whose genius may qualify them for the herculean task.

